# Aeolian process effects on vegetation communities in an arid grassland ecosystem

**DOI:** 10.1002/ece3.205

**Published:** 2012-04

**Authors:** Lorelei J Alvarez, Howard E Epstein, Junran Li, Gregory S Okin

**Affiliations:** 1 Department of Environmental Sciences, University of VirginiaClark Hall, 291 McCormick Road, Charlottesville, VA 22904-4123; 2 Department of Geography, University of California1255 Bunche Hall, Box 951524, Los Angeles, CA 90095

**Keywords:** Aeolian processes, arid grassland, Chihuahuan desert, community changes, sediment flux, shrub expansion

## Abstract

Many arid grassland communities are changing from grass dominance to shrub dominance, but the mechanisms involved in this conversion process are not completely understood. Aeolian processes likely contribute to this conversion from grassland to shrubland. The purpose of this research is to provide information regarding how vegetation changes occur in an arid grassland as a result of aeolian sediment transport. The experimental design included three treatment blocks, each with a 25 × 50 m area where all grasses, semi-shrubs, and perennial forbs were hand removed, a 25 × 50 m control area with no manipulation of vegetation cover, and two 10 × 25 m plots immediately downwind of the grass-removal and control areas in the prevailing wind direction, 19° north of east, for measuring vegetation cover. Aeolian sediment flux, soil nutrients, and soil seed bank were monitored on each treatment area and downwind plot. Grass and shrub cover were measured on each grass-removal, control, and downwind plot along continuous line transects as well as on 5 × 10 m subplots within each downwind area over four years following grass removal. On grass-removal areas, sediment flux increased significantly, soil nutrients and seed bank were depleted, and *Prosopis glandulosa* shrub cover increased compared to controls. Additionally, differential changes for grass and shrub cover were observed for plots downwind of vegetation-removal and control areas. Grass cover on plots downwind of vegetation-removal areas decreased over time (2004–2007) despite above average rainfall throughout the period of observation, while grass cover increased downwind of control areas; *P. glandulosa* cover increased on plots downwind of vegetation-removal areas, while decreasing on plots downwind of control areas. The relationships between vegetation changes and aeolian sediment flux were significant and were best described by a logarithmic function, with decreases in grass cover and increases in shrub cover occurring with small increases in aeolian sediment flux.

## Introduction

Many arid grassland ecosystems are currently undergoing major community shifts from grass-dominated vegetation to shrub-dominated habitat. Declines in grass species, increases in bare ground area, increases in wind erosion, and loss of soil nutrients are commonly associated with these changing communities (Schlesinger et al. 1990; Gillette and Pitchford 2004; Peters et al. 2006; Li et al. 2007); although, recent research results demonstrate that not all changes associated with shrub expansion are negative and largely depend on the particular region studied as well as the variables used to indicate ecosystem function (Maestre et al. 2009; Eldridge et al. 2011). Much research has focused on arid grassland degradation and invasion of woody species, and a variety of hypotheses have been published to explain causes and mechanisms involved in this transition (see Schlesinger et al. 1990, 2000; Archer et al. 1995; Larney et al. 1998; Van Auken 2000; Okin et al. 2001a, b, 2009a, b; Geist and Lambin 2004; Laliberte et al. 2004; Briggs et al. 2005; Gibbins et al. 2005; Browning et al. 2008). Whether these changes should be considered negative, as many researchers have suggested, or not necessarily negative and dependent on the measures of ecosystem function in question (Eldridge et al. 2011), it is still important to understand the process of conversion and the mechanisms involved in shrub expansion.

Researchers focusing on the causes of shrub expansion believe that multiple factors are involved in this transition, but there is a lack of consensus as to which factor(s) are the most important. Brown et al. (1997) reported finding that shrub expansion in the southwestern United States is correlated with regional climate changes, specifically with an increase in winter precipitation for the region. Van Auken (2000) and Briggs et al. (2005) reported fire suppression in combination with overgrazing as the main factors involved in this conversion. Some researchers report that drought and overgrazing are likely the most important causes (Geist and Lambin 2004). Other researchers have reported drivers such as microclimatic modification by shrubs (He et al. 2010) and rodent herbivory (Bestelmeyer et al. 2007). Peters et al. (2006) report that the dynamics of these ecosystems are actually much more complex, and at least five different factors need to be considered in order to be able to predict long-term vegetation changes. They suggest that it is necessary to consider (1) historical legacies, including climate changes and past disturbances; (2) environmental driving factors, including current weather patterns and disturbances; (3) soil geomorphology; (4) transport vectors (wind, water, and animals) for soil nutrients and seed bank; and (5) resource redistribution. This is an important conceptual framework for understanding the complexity of these community changes, but it is also necessary to understand how and why each of these factors contributes to community changes.

Schlesinger et al. (1990) proposed that the conversion from grassland to shrub-dominated habitat occurs through the redistribution of soil resources from a homogeneous distribution to a heterogeneous distribution, with water and nutrients concentrating beneath shrub canopies. Initial reduction of grasses (as occurs with extensive grazing) leads to decreased rainfall infiltration rates, increased runoff, and accumulation of water and other resources beneath shrubs, which in turn leads to increases in shrub cover. The redistribution of soil resources from increased runoff, creation of “islands of fertility” beneath shrub canopies, and changes in hydrology and infiltration rates during grassland transition to shrubland have been well documented, particularly in the arid/semiarid grasslands of the United States (Crawford and Gosz 1982; Abrahams et al. 1995; Schlesinger et al. 1990, 1996; Hook et al. 1991; Franzluebbers et al. 2000; D’Odorico et al. 2007; Pockman and Small 2010; Ravi et al. 2010; Turnbull et al. 2010) However, Schlesinger et al. (2000) also reported that water erosion alone cannot account for the redistribution of soil nutrients beneath shrub canopies. Okin et al. (2009) built on the Schlesinger et al. (1990) fertile island model for woody encroachment by suggesting that the changes in connectivity, in this case, for the movement of aeolian and fluvial sediments, is responsible for the maintaining the positive feedbacks that are responsible for the stability of shrub-dominated systems.

In many arid environments, water-based transport of soil nutrients is limited due to closed basins and fairly uniform, flat topography (Gillette and Pitchford 2004). Okin et al. (2001) proposed that much of the redistribution of soil resources can actually be explained by wind erosion (as opposed to water erosion) as a result of increased wind erosion rates associated with these shifting vegetation communities. Aeolian transport is an important process in arid environments, and recent research indicates that removal of grasses in these sparsely vegetated ecosystems can greatly increase the wind erosion rates and reduce soil nutrient stores (Li et al. 2007, 2008). On treatment plots, where grasses had been removed, Li et al. (2007) observed a 15% reduction in total organic carbon (TOC) and total nitrogen (TN) from the top 5 cm of soil in just one windy season, and a reduction of 25% after three windy seasons. Additional research results indicate that soil nutrients are not only depleted, but redistributed through the disappearance of smaller clusters of nutrients related to grass locations, and the increase of large “fertile islands” associated with shrub locations (Li et al. 2008). These results suggest that wind erosion plays a crucial role in these ecosystems, potentially removing soil, depleting soil nutrients, and increasing soil nutrient heterogeneity.

A recent review of the literature indicates that redistribution of soil resources and shrub expansion into grasslands is likely the result of the combined effects of water and wind erosion, but that aeolian processes may dominate in more arid regions (Ravi et al. 2010). Okin et al. (2006) proposed that the redistribution of soil resources resulting from grass reduction and wind erosion will abruptly shift the system to a shrub-dominated landscape with soil nutrients and seed bank stores depleted in intershrub areas, and that this converted shrubland will be a stable state that is no longer able to support grassland. We further propose that disturbance followed by increased wind erosion, and depletion and redistribution of soil nutrients and the soil seed bank also leads to a shifting vegetation community downwind of the initial point of disturbance.

**Figure 1:**
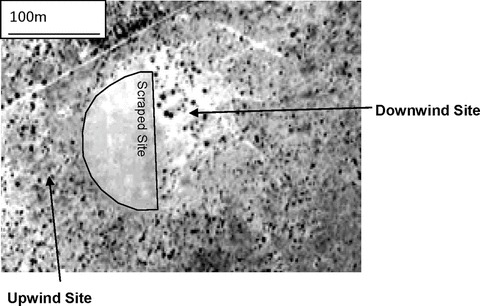
Satellite imagery of an area on the Jornada Experimental Range, where all vegetation was removed and the surface scraped bare to promote wind erosion “scraped site.” The image shows the scraped site as well as adjacent upwind and downwind areas (“downwind site” and “upwind site”). The lighter areas shown downwind of the site are bare soil areas with patchy shrub coverage, whereas the grayer areas shown upwind are covered with grass.

**Figure 2:**
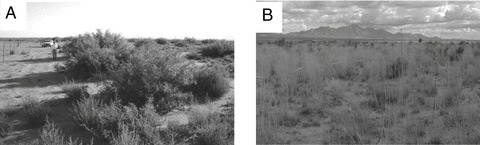
Vegetation downwind of the scraped site (A) versus upwind of the scraped site (B). The downwind image was taken immediately adjacent to the scraped site in the prevailing wind direction. The upwind image was taken approximately 50 m from the scraped site in the upwind direction.

Changes in vegetation communities downwind of areas of increased wind erosion have been observed at the Jornada Experimental Range (JER) in south-central New Mexico (Okin and Gillette 2001a). A large area of the JER was cleared of vegetation in 1990. Vegetation has not been allowed to regrow on the area over the past 20 years, and although intense data collection was not conducted on the area downwind of the site, observations from satellite imagery indicate major shrub expansion and grass cover reduction adjacent to the scraped site in the prevailing wind direction (Fig. 1). Both satellite imagery and in situ observations show that shrub cover is much greater downwind of the scraped site in the prevailing wind direction, while grass cover is greater than shrub coverage on the adjacent upwind areas (Figs. 1 and 2a, b). Okin et al. (2001a) also reported significant nutrient depletion in both the erosional and depositional areas.

Based on the conceptual models proposed by Okin et al. (2001a, b, 2009) and assessed with computational simulations (Okin et al. 2009), observations of community changes downwind of the scraped site at the JER (Okin and Gillette 2001), as well as research on soil nutrient depletion and redistribution (Okin et al. 2001a; Li et al. 2007, 2008), it seems clear that wind erosion can be involved in shrub expansion in arid grasslands as follows: (1) Some initial disturbance reduces grass cover (e.g., overgrazing or drought); (2) This grass removal leads to increased size of bare gaps that serve as connected pathways for aeolian transport resulting in aeolian flux rates as well as a release for the shrubs from competition for belowground resources; (3) At the surface, the increased wind erosion depletes soil nutrients and the seed bank, leading to shrub dominance in disturbed areas; (4) Much of the eroded material is deposited over short distances downwind of the initial disturbance; (5) The deposition tends to be concentrated beneath shrub canopies, allowing for shrub expansion due to increased nutrient availability and perhaps burial and/or abrasion of grasses; (6) Eventually the area downwind also becomes shrub-dominated with large bare gaps and high connectivity, wind erosion increases, soil nutrients and seed bank are depleted, and the entire process is repeated and “moves” downwind from the point of initial disturbance. Any further disturbances, such as continuing drought or overgrazing, would likely serve to enhance these effects, increase loss of soil resources, and possibly decrease the overall time necessary for the conversion process to occur.

We established an experiment in a grass-dominated pasture, interspersed with a few small shrubs, at the JER in order to test aspects of our overall hypothesis for shrub expansion in arid grasslands. The experiment allowed us to specifically test whether (1) grass removal leads to increased wind erosion, and soil nutrient and seed bank reduction (reported inLi et al. 2007, 2008); (2) shrub expansion occurs as a result of that initial disturbance; (3) redistribution of soil nutrients beneath shrub canopies occurs in adjacent, downwind areas (reported here and in Li et al. 2008); and (4) shrub cover increases and grass cover decreases on the downwind areas. Here, we report mainly on the impact of the experiment on vegetation both on and downwind of the disturbance (2 and 4 above).

## Materials and Methods

### Study area

We established our experiment at the Jornada Experimental Range (JER). The JER is an approximately 783 km^2^ arid-grassland/shrubland site located in south-central New Mexico in the Chihuahuan Desert. The region has a mean annual rainfall of less than 250 mm, with more than 50% of this precipitation occurring during July through September, mean maximum summer temperatures of 36°C, and mean maximum winter temperatures of 13°C (data available from JER). Major increases in shrub cover (particularly in mesquite dune habitat) and declines in once abundant grasses such as *Bouteloua* spp. and *Sporobolus* spp. have occurred over the last 100–150 years (Buffington and Herbel 1965; Laliberte et al. 2004; Gibbins et al. 2005).

Our experiment was established in Pasture 11 of the Long Term Ecological Research (LTER) site (32°56′N, 106°75′W) on what is commonly called the “sand sheet.” Soils at the site generally have a loamy sand to sandy loam texture. Seventy-nine percent of the winds occur in a southwesterly direction, and dominant wind erosion events occur during early March to May (Helm and Breed 1999). Treatment areas were selected for high grass cover, low shrub cover, and fairly uniform topography and high infiltration rates (to minimize runoff effects), and were located near each other so that soil texture and nutrient levels, precipitation, and wind regimes were all similar.

### Experimental design and data collection

**Figure 3:**
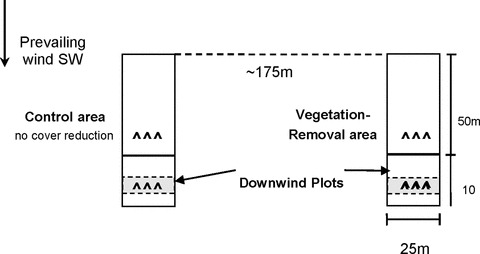
Example of the experimental layout. Three of these treatment areas or blocks were included in the study. Each block included a 25 × 50 m plot where 100% of grasses, semishrubs, and perennial forbs were manually removed (removal area), a 25 × 50 m area with no vegetation manipulation (control area), and adjacent, 15 × 25 m plots downwind of the vegetation-removal and control areas. On each downwind plot, three, 10 m continuous line transects were used to measure vegetation cover (2004–2007). A 5 × 10 m vegetation distribution plots were also established 5 m from the downwind edge of the vegetation-removal and control areas (placement indicated by light gray squares). “^⁁^” symbols represent approximate placement of dust collectors for monitoring dust flux. Soil samples for nutrient analysis were collected within 5 m of the upwind edge of the control, treatment, and downwind plot. Samples for seed bank germination were collected approximately 15 m from the downwind edge of the treatment and control area and within 5 m of the upwind edge of the downwind plot.

The experimental design included three treatment areas or blocks, each including the following: a 25 × 50 m area where all grasses, semishrubs such as *Gutierrezia sarothrae*, and perennial forbs were hand removed (hereafter referred to as “grass-removal areas”); a 25 × 50 m “control area” with no manipulation of vegetation cover; two 25 × 15 m plots immediately downwind of the vegetation-removal and control areas in the prevailing wind direction (“downwind plots”); and two 5 × 10 m plots, 5 m from the downwind edge of the vegetation-removal and control areas (“vegetation distribution plots”; Fig. 3). The dominant grasses on two of the three blocks were *Sporobolus flexuosus*, while the dominant grass in the third block was *Bouteloua eriopoda*. Manual removal of grasses was conducted using gas-powered weedeaters in July 2004 and maintained through 2007; *Prosopis glandulosa* shrubs were not removed.

To monitor dust flux on grass-removal areas, control areas, and downwind plots, Big Spring Number Eight (BSNE) aeolian samplers were installed in the spring and summer of 2004. These collectors were developed by Fryrear (1986) and have been independently tested by Shao et al. (1993). Detailed BSNE setup was described in Li et al. (2007). Six samplers (two rows of three) were placed on each grass-removal and control area: one row ∼25 m and a second row ∼35 m from the upwind edge (Fig 3). Six collectors were also placed on the downwind plots, one row ∼5 m and one row ∼15 m from the downwind edge of the grass-removal and control areas (Fig. 3). Samplers were attached to a rotating wind vane, mounted at different heights on a central pole. The samplers were placed 3–4 m apart, perpendicular to the prevailing wind, while avoiding placement directly downwind of shrubs. On each treatment, control area, and downwind plot, samplers were placed at heights 0.1, 0.3, 0.6, and 1.2 m above the ground. Traps were emptied just before and after each windy season (March–May) from 2004 –to 2006. The amount of sediment collected during each windy season was weighed to 0.001 g, and the mass used to calculate aeolian sediment flux for each area (Li et al. 2007).

Six soil samples were collected to a depth of 10 cm from each treatment, downwind, and control plot within 5 m of the windward edge (Fig. 1). Three of the six samples were collected from bare areas between plants, while the remaining three were collected from beneath shrub canopies. Soil samples were collected during the establishment of the experiment in 2004 and repeated in 2006 and used to analyze soil total organic carbon (TOC) and total nitrogen (TN).

On each grass-removal and control area, we used a Trimble 3600 total station (Trimble Navigation Limited, Sunnyvale, CA) to record the locations of all perennial plants (only *P. glandulosa* after grass removal), and we measured widths parallel and perpendicular to the prevailing wind direction for each. Location and plant dimension data were also recorded on the 5 × 10 m downwind, vegetation distribution plots for all species. Total station coordinate data and plant dimensions for blocks 1 and 3 were collected during the summer of 2004. Block 2 data were collected in July 2005. Measurements for all plots were repeated during the summer of 2007. Vegetation cover was also measured on each downwind plot using three, continuous line transects per plot. Each grass, shrub, semishrub, and perennial forb was identified, and its length parallel to the prevailing wind direction recorded. Cover data along transects were recorded during 2004 prior to grass removal and each year posttreatment, 2005–2007.

Additional soil samples were collected to analyze the effects of grass removal on the soil seed bank in both grass-removal and adjacent, downwind plots and compared to control areas for two years following treatment initiation (2005 and 2006). We collected 10 samples (five from under shrub canopies and five in bare spaces between plants) from each grass-removal and control area approximately 15 m from the downwind edge, and 10 samples approximately 5 m from the windward edge of the downwind plots (Fig. 3). Samples were 6-cm diameter by 2-cm depth, as previous research at the JER has observed the largest seed concentrations in the top 2 cm of soil (Goodall and Morgan 1973; Guo et al. 1998). We sifted samples to remove large organic debris and then germinated seeds to determine the number of viable seeds in each sample using the methods ofGross (1990). Soil samples for seed bank analysis were spread in 2-cm-thick layers and germinated under full-spectrum fluorescent growth lights set on timers to mimic the number of daylight hours typical of the JER during the growing season. Samples were kept moist throughout the germination period. All seedlings that emerged were counted and identified by plant type (grass or forb). Germinated seedlings typically did not live long enough to identify the species. Germination was conducted during the fall after sample collection for both years.

### Laboratory and statistical analyses

Soil samples collected for nutrient analyses were air-dried, sieved to 2 mm, and then ground to a fine power by a ball mill (Model 2601Cianflone, Scientific Instruments Corporation, Pittsburgh, PA). Five gram subsamples were then used for analysis of TOC and TN. Vanadium oxide (V_2_O_5_) was used as a combustion catalyst with the mass ratio of catalyst to sample of approximately 1:1 to ensure complete decomposition of all carbonates in TC measurements. TOC and TN analysis was conducted on a Shimadzu TOC-VCSN TOC analyzer with a SSM-5000 solid sample analyzer and a TNM-1 TN measuring unit (Shimadzu Scientific Instruments Inc., Columbia, MD). Analysis of variance (ANOVA) was used to determine whether TOC and TN levels changed from 2004 to 2006 across treatments for samples collected beneath shrub canopies and in open spaces.

Fractional cover of grasses (all species), *P. glandulosa*, *G. sarothrae*, and perennial forbs (all species) for the downwind plots were calculated by summing the total length covered and dividing by 10 m, the length of the transect. Percent bare ground area was calculated by subtracting the total ground covered by vegetation from 10 m and dividing by 10 m. Fractional cover of *P. glandulosa* shrubs on the grass-removal and control areas, and grasses and shrubs on each of the downwind, vegetation distribution plots was also calculated as the sum of the area of the plants (assuming circular-shaped grasses and shrubs), divided by total plot area.

ANOVA was used to determine whether shrub cover changed over time for grass removal versus controls, and whether cover for grasses, shrubs, semishrubs, and forbs changed over time downwind of grass-removal and control areas. Percent cover values were transformed using arcsine square-root transformations in order to meet the assumptions for ANOVA. Percentage changes in vegetation cover for grasses and shrubs were also calculated based on pretreatment values (2004 coverage values), and regression analyses were used to examine relationships between these changes and dust flux (*Q*) from the previous windy season.

The number of seedlings germinated from samples collected on treatment, control, and downwind areas for the two years was also compared using repeated-measures ANOVA. As differences in the numbers of grasses versus forbs that emerged were not observed, total number of seedlings was used for this analysis. Seedling counts were transformed using Log *x*+ 1 transformations to ensure data fit assumptions for ANOVA.

## Results

**Figure 4:**
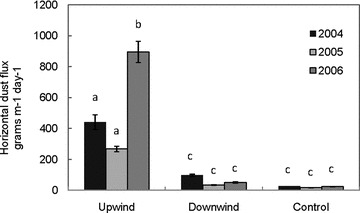
Horizontal dust flux (g·m^−1^·day^−1^) across treatments with each year shown separately. Across all years, there was significantly more dust flux on the vegetation-removal treatments than the downwind or control treatments (*P*≤ 0.05). Lower-case letters above error bars show significant differences as demonstrated by LSD post-hoc analysis.

**Figure 5:**
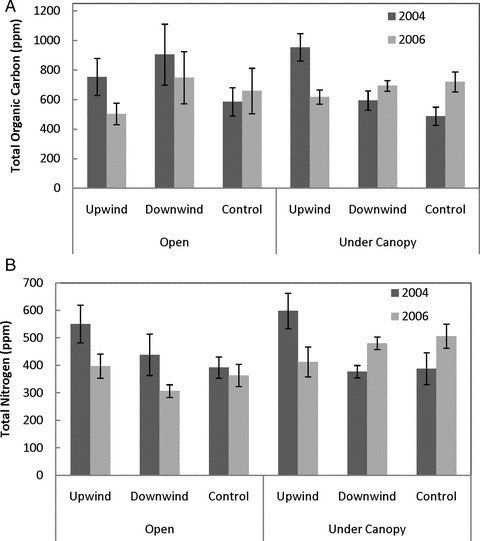
Total organic carbon (A) and total nitrogen (B) parts per million (PPM) ± standard error for control, grass-removal (upwind), and unmanipulated, downwind plots for samples collected under shrub canopies and in open spaces between plants across two years of observation (2004 and 2006). For TN, a significant three-way interaction was observed, while for total organic carbon, a significant treatment by year interaction was observed.

Across all three years that dust flux was monitored, there was significantly greater horizontal dust flux (g·m^−1^·day^−1^) on the vegetation-removal treatment area than the control area or downwind or plots (*P* < 0.01) (Fig. 4). Horizontal dust flux was not significantly different between the downwind plots and control areas. For soil nutrient analysis, TN levels were lower in 2006 than 2004 for vegetation-removal areas and downwind plots for samples collected in open spaces, while levels for samples collected from control areas did not change. For samples collected under shrub canopies, nutrient levels were lower in 2006 for vegetation-removal areas only, while levels increased under shrub canopies for downwind plots and control areas (significant three-way interaction; *P*= 0.05; Table 1; Fig. 5a, b). Similar patterns were observed for TOC levels, but the interaction was only significant for treatment by year (with decreased levels on vegetation-removal areas only; Table 1; Fig. 5a, b).

**Table 1:** (A) Analyses of variance (ANOVA) results for dust flux and vegetation measures of shrub, grass, semishrub, forb cover, and number of grasses per year of observation across treatments (grass removal, downwind, and control). (B) ANOVA results for soil nutrients (total organic carbon [TOC] and total nitrogen [TN]) and soil seed bank analyses per year of observation, across treatments, and under shrub canopies versus open spaces.

	Year		Treatment		Year × treatment	
	*F*	Significance	*F*	Significance	*F*	Significance
(A)						
Dust flux	5.15	0.02	21.08	0.00	4.76	0.02
Removal shrub cover (TS)	25.85	0.01	1.63	0.27	9.43	0.04
Downwind shrub cover (TS)	0.40	0.56	1.96	0.23	22.56	0.01
Downwind grass cover (TS)	0.68	0.46	0.73	0.44	6.95	0.05
Number of grasses downwind	5.72	0.08	0.89	0.40	0.64	0.46
Downwind shrub cover (transect)	0.25	0.86	1.66	0.21	1.60	0.21
Downwind grass cover (transect)	3.14	0.06	4.20	0.05	5.52	0.01
Downwind semishrub cover (transect)	18.69	0.00	7.21	0.01	3.53	0.05
Downwind forb cover (transect)	34.36	0.00	0.06	0.80	2.28	0.10
(B)						
Soil TOC	1.18	0.29	0.46	0.64	6.47	0.01
Soil TN	2.68	0.11	1.75	0.19	5.01	0.01
Seedlings germinated	0.45	0.84	11.10	0.00	0.32	0.74

	Canopy		Treatment × canopy		Year × treatment × canopy	
	*F*	Significance	*F*	Significance	*F*	Significance

Soil TOC	0.02	0.90	0.63	0.54	0.87	0.43
Soil TN	1.69	0.21	0.08	0.92	4.20	0.05
Seedlings germinated	12.14	0.01	4.40	0.04	0.32	0.74

**Figure 6:**
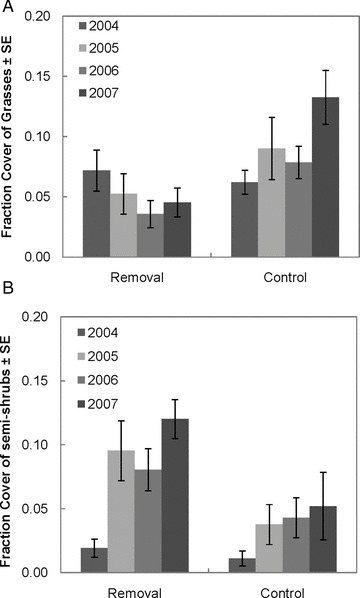
Fractional coverage of vegetation along continuous line transects covering 10 m immediately downwind of the vegetation-removal and control areas. (A) Fractional grass cover. (B) *Gutierrezia sarothrae—*semishrub cover. Significant treatment by year interactions were observed for both.

Shrub cover was significantly higher in 2007 than in 2004 in the vegetation-removal areas, but was not different for control areas (significant year by treatment interaction; Table 1). Data from line transects indicate that grass cover on plots downwind of vegetation-removal areas was lower in 2007 than in 2004, but was higher in 2007 than initial data collection in 2004 for plots downwind of control areas (significant year by treatment interactions; *P*≤ 0.05; Table 1; Fig. 6). *Gutierrezia sarothrae* cover was higher on plots downwind of both grass-removal and control areas in 2007 than in 2004, but the change in cover was greater for plots downwind of the grass-removal areas (Fig. 6a, b). Perennial forb cover did change over time, but there was no significant interaction between year of observation and treatment. Total vegetation cover values (all species) for plots downwind of vegetation-removal and control areas were similar, and values were lower during the summer of 2006 than all other years of data collection. Percentage of bare ground was not significantly different over time or between plots downwind of vegetation-removal or control areas.

**Figure 7:**
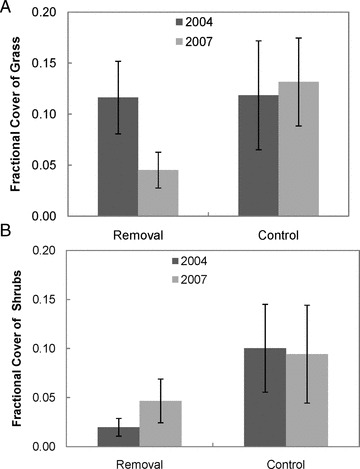
Fractional cover of (A) grasses and (B) mesquite shrubs on the 5-m vegetation distribution plots. “Removal plot” was located 5-m downwind of the vegetation-removal area and “control plot” was 5-m downwind of the area with no vegetation removed. Statistical analyses demonstrated significant (*P*≤ 0.05) interactions between treatment plots and year of observation for both grass and shrub cover.

Significant interactions between year of observation and treatment were observed for grass and *P. glandulosa* shrub cover calculated using the 5 × 10 m vegetation distribution plots downwind of grass-removal and control areas (Table 1). Grass cover was higher in 2007 than in 2004 downwind of controls, and was slightly lower in 2007 than in 2004 downwind of grass-removal areas. Shrub cover was higher in 2007 downwind of grass-removal areas than in 2004, but did not change from 2004 to 2007 downwind of control areas (Fig. 7a, b). We also counted the number of grasses on each plot in 2004 and again in 2007. The number of grass individuals decreased from 2004 to 2007 (approaching significance; Table 1), indicating grass mortality, and this decrease was observed for plots downwind of both vegetation-removal and control areas (no significant interaction).

**Figure 8:**
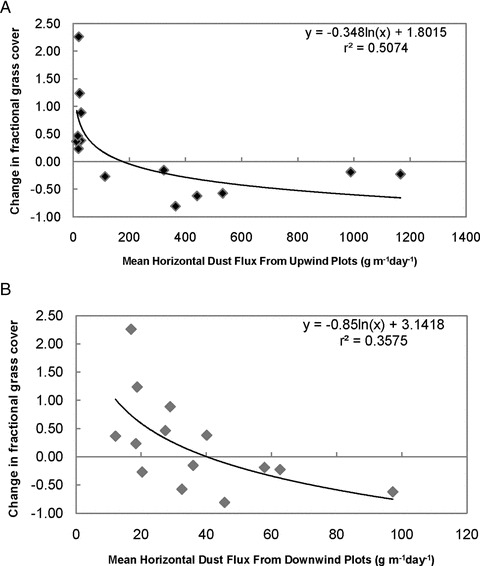
Proportional changes from pretreatment levels in fractional grass cover downwind of vegetation-removal and control areas as a function of dust flux from the preceding windy season. (A) Vegetation changes on the adjacent, downwind plots as a function of aeolian sediment flux from the vegetation-removal and control areas. (B) Cover changes as a function of aeolian sediment flux on the downwind plots. Both show significant logarithmic relationships, but more of the variation in grass cover change is explained by the dust flux from the upwind vegetation-removal and control areas (*r*^2^= 0.5).

**Figure 9:**
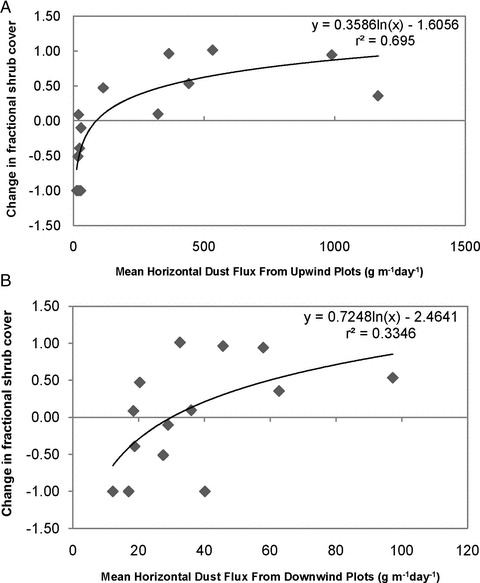
Proportional changes from pretreatment levels in fractional shrub cover downwind of vegetation-removal and control plots as a function of dust flux from the preceding windy season. (A) Shrub cover changes on downwind plots as a function of dust flux from the vegetation-removal and control areas. (B) Shrub cover changes as a function of dust flux on the downwind plots. Both show significant logarithmic relationships, but more of the variation in shrub cover change is explained by the dust flux from the upwind vegetation-removal and control areas (*r*^2^= 0.7).

Regression analyses were used to examine relationships between horizontal aeolian sediment flux for vegetation-removal, control, and downwind plots for each windy season preceding vegetation data collection and the percent changes in grass and *P. glandulosa* cover for each year of data collection (2005–2007) from pretreatment values (2004). Results of these analyses demonstrated that (1) grass cover decreases as dust flux increases (significant logarithmic relationships, Fig. 8a and b) with the logarithmic fit crossing zero change in grass cover at ∼175 g·m^–1^·d^–1^ (flux measured on upwind plots) or ∼40 g·m^–1^·d^–1^ (flux measured on downwind plots); and (2) shrub cover increases as dust flux increases (significant logarithmic relationship, Fig. 9a and b, with the logarithmic fit crossing zero change in shrub cover at ∼90 g·m^–1^·d^–1^ (flux measured on upwind plots) or ∼30 g·m^–1^·d^–1^ (flux measured on downwind plots)).

**Figure 10:**
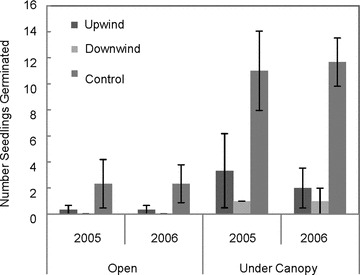
Average number seedlings germinated (±SE) per sample collected under shrub canopies and in open spaces, separated by treatment (upwind = grass removal, downwind = adjacent downwind area, and control) for both years of sample collection. The greatest number of seedlings was germinated from samples collected under shrub canopies on control treatments (significant treatment by location interaction).

ANOVA results demonstrated that significantly more seedlings were germinated from samples collected under shrub canopies than in open spaces between plants, and also for control areas compared to treatment and downwind plots for both 2005 and 2006 (Table 1; Fig. 10). The greatest number of seedlings was germinated from samples collected under shrub canopies on control treatments (significant treatment × location interaction).

## Discussion

Shrub expansion in arid grasslands is well documented, and it is also clear that there are many factors involved in this transition including past disturbances, current disturbances, global climate changes, local and regional climate patterns, and redistribution of resources through water and wind transport (Peters et al. 2006; Ravi et al. 2010). However, the mechanisms involved in this conversion process and the amount of change that can be accounted for by each of these different factors is not well understood. The purpose of this research was to provide additional information regarding one of the potential mechanisms of conversion, wind transport. We designed this experiment to test our hypothesis regarding the involvement of wind transport in shrub expansion in arid grasslands. Our results provide strong support for that overall hypothesis, each component of which is discussed below.

We manually removed grass cover as would occur with extensive overgrazing in a pasture. This removal led to increased bare ground area with larger unvegetated gaps and increased wind transport rates. We observed significantly more wind transport on grass-removal areas than on control areas. The increased wind transport depleted soil nutrients and seed bank, presumably decreasing the chance for grass reestablishment in interplant spaces, and leading to shrub dominance in disturbed areas. Levels of TOC and TN decreased on vegetation-removal plots (demonstrated by both our nutrient analyses and previous research on these plots; Li et al. 2007). The number of seedlings germinated from soil samples was significantly lower for samples collected from grass-removal areas and for samples collected from bare-surface areas than from the control areas. This reduction was observed to occur just one year after grass removal, indicating that the soil seed bank is depleted rapidly with increased aeolian transport. Shrub cover also increased significantly on grass-removal areas compared to control areas, presumably due to release of the shrubs from competition with grasses for belowground resources.

Much of the eroded material is deposited over short distances downwind of the initial disturbance, as indicated by much lower rates of transport on the downwind plots compared to the vegetation-removal plots (see Li et al. 2008). Levels of TOC and TN decreased in downwind areas in spaces between plants, but actually increased over the three-year period of observation for soil samples collected under shrub canopies (significant interaction for TN only). These results are consistent with previous research showing concentration of nutrients under shrub canopies in areas affected by increased aeolian sediment transport (Li et al. 2008). We observed decreased grass cover and increased shrub cover downwind of grass-removal areas across all blocks and all years of data collection, and increased grass cover and a decreased shrub cover downwind of control areas from 2004 to 2007. Minor differences were observed between transect cover data and cover data calculated from the vegetation distribution plots, but those differences could be caused by spatial heterogeneity or because of the more detailed measurements involved in the vegetation distribution plots. Despite these slight differences, both transect data and vegetation distribution data indicate that grass cover decreases and shrub cover increases downwind of disturbed areas with increased wind erosion. The only different controlling factor between the plots downwind of the grass-removal and control areas was the increased wind transport rates on the areas where vegetation had been removed. The flat topography of the sites, high infiltration rates on sandy soils, and lack of any geomorphic evidence of large-scale fluvial erosion, indicates that our observations are likely not related to transport of sediments by water. Additionally, the placement of the plots used for monitoring vegetation changes was immediately downwind of the grass-removal and control areas in the prevailing wind direction, which provides support for these changes resulting from the increased wind erosion and soil nutrient redistribution.

The actual number of grasses on the vegetation distribution plots was lower in 2007 than 2004 downwind of both grass-removal and control areas, indicating grass mortality across treatments; however, decreased grass cover was observed only in the plots downwind of the grass-removal areas. Grass mortality downwind of control areas could be the result of one or a combination of the many other factors linked to community changes in these ecosystems, including fire suppression, past disturbances, or climate changes (Geist and Lambin 2004; Briggs et al. 2005; Peters et al. 2006). However, the grasses remaining on the plots downwind of the control areas increased in size so that the area covered by grasses either stayed the same or increased slightly, while the area covered by grasses decreased downwind of the vegetation-removal areas.

Another interesting observation was that the percentage of bare ground was not different between plots downwind of grass-removal or control areas and did not change over time. This is consistent with results reported by Eldridge et al. (2011) and indicates that although the vegetation composition did change for plots downwind of vegetation-removal areas, those changes were not associated with an increase in bare ground area (one of the suggested negative outcomes of the process of grassland conversion to shrubland). Grass cover decreased, while shrub and semishrub (*G. sarothrae)* cover increased. The results of the regression analyses gave additional information regarding the relationship between vegetation changes over time and dust flux. Our results indicate that there are significant relationships between the amount of aeolian sediment flux into an area and declines in grass cover, and between the amount of aeolian sediment transport and increases in shrub cover. Small increases in sediment flux can lead to relatively large changes in vegetation cover for both grasses and shrubs. Sediment flux on areas upwind of plots where vegetation changes were observed accounted for more of the vegetation change (both for grass decreases and shrub increases) than dust flux on the actual plots where the vegetation changes occurred. This also provides support for our hypothesis and for the conceptual model proposed by Okin et al. (2001a, b) that the vegetation changes occur downwind as a result of the wind transport and deposition from upwind. According to our regression analyses, about 50% of the changes in grass coverage and 70% of the changes in shrub coverage can be accounted for by the flux from the treatment areas upwind of the observed changes.

Our observations show that eventually the area downwind may become shrub dominated, with accompanying increases in wind transport (Gillette and Pitchford 2004) and decreases in soil nutrients and seed bank (this study and Li et al. 2007). This provides support for a positive feedback in which the entire process can be supported in additional areas downwind. The results of our research indicate that this process actually begins relatively quickly after an initial disturbance and is consistent with modeling results indicating that removal of grasses will rapidly shift these grass-dominated ecosystems to a stable shrub-dominated habitat that is no longer able to support grasslands (Okin et al. 2009b).

## Conclusion

Our results, in combination with the research results presented by Li et al. (2007, 2008) provide a better understanding of the process of shrub expansion into previously grass-dominated habitat, and provide support for our overall hypothesis of the mechanisms involved in shrub expansion for this particular system. Given these results, we suggest that shrub expansion in arid grasslands occurs as follows: grasses and shrubs directly compete for resources in areas where grasses dominate. Grass removal from some initial disturbance, such as overgrazing or drought gives shrubs greater access to resources and increases shrub cover. This grass removal also results in increased aeolian flux rates, depletion and redistribution of soil nutrients, and depletion of the soil seed bank (this study and Li et al. 2007, 2008). Much of the eroded material is deposited over short distances downwind of the initial disturbance, and tends to be concentrated beneath shrub canopies (this study and Li et al. 2008), allowing for shrub expansion possibly due to increased nutrient availability. Eventually the area downwind also becomes shrub-dominated, wind erosion increases, soil nutrients and seed bank are depleted, and the entire process is repeated and “moves” downwind from the point of initial disturbance. We further propose that this same process would likely occur for any arid grassland ecosystem where shrubs are present and there is a strong prevailing wind direction; however, research in similar ecosystems would need to be conducted to determine whether these results can indeed be generalized globally.
